# Mechanisms of Color Production in a Highly Variable Shield-Back Stinkbug, *Tectocoris diopthalmus* (Heteroptera: Scutelleridae), and Why It Matters

**DOI:** 10.1371/journal.pone.0064082

**Published:** 2013-05-07

**Authors:** Scott A. Fabricant, Darrell J. Kemp, Jan Krajíček, Zuzana Bosáková, Marie E. Herberstein

**Affiliations:** 1 Department of Biological Sciences, Macquarie University, North Ryde, New South Wales, Australia; 2 Department of Analytical Chemistry, Charles University in Prague, Prague, Czech Republic; Arizona State University, United States of America

## Abstract

Theory suggests that aposematism, specifically the learned avoidance of unprofitable prey via memorable color patterns, should result in selection for pattern uniformity. However, many examples to the contrary are seen in nature. Conversely, honest sexual signals are likely to exhibit greater variation because they reflect underlying variation in mate quality. Here we aim to characterize and quantify the mechanistic causes of color in *Tectocoris diopthalmus* to shed light on the costs of color production, and thus the potential information content of its color signals. We use *Tectocoris diopthalmus* because it is a weakly-defended stinkbug, and presents elements that have classically been studied in the context of aposematism (red coloring), and sexual selection (sexual dichromatism and iridescent coloring). Pigment analysis reveals that variation in orange coloration is due to the amount of erythropterin pigment, stored in intracellular granules. This pigment is common in Heteroptera, and as an endogenously produced excretory byproduct is unlikely to reflect mate quality or variation in unprofitability of the bug. Electron microscopy reveals the iridescent patches are caused by an epicuticular multilayer reflector, and the hue and patch size are directly related to the layer widths and extent of coverage of this layering. Furthermore, we identified melanin as an essential component of the multilayer reflector system; therefore, the quality of the iridescent patches may be affected by aspects of rearing environment and immunocompetence. We posit that *T. diopthalmus* has co-opted the melanic patches of a ‘typical’ red and black aposematic signal, transforming it into a complex and variable iridescent signal that may enhance its capacity to display individual quality.

## Introduction

Aposematism is the phenomenon wherein defended prey advertise unprofitability through conspicuous signals. Often these take the form of bright ‘warning colors’, as it is theorized that conspicuous coloration aids predator recognition and memory [1]. Generally, the ‘typical’ aposematic patterns of insects are red or yellow coloration with black markings [2] [3].

Theory suggests that aposematic patterns should be under strong frequency-dependent selection by predators to be monomorphic [4] [5], and/or strong directional selection for conspicuousness [6] [7]. However, examples of species with variable patterns or other deviations from theoretical predictions are common [3]. Recent theoretical treatments have addressed possible causes of intermediate aposematism and intraspecies variation in pattern, via such mechanisms as predator community structure [8], or variation in prey defense levels [9]. Notably, Blount's et al. [10] model of intraspecific variation explicitly explores color production mechanisms as drivers of variation, through the vehicle of pigments acting as antioxidants to defend against autotoxicity. Recent empirical studies have also demonstrated possible trade-offs between warning signal quality and physiological traits such as toxin excretion costs [11] and pathogen resistance [12], It is hence possible than the mechanisms of aposematic signals themselves, and related physiological traits, are contributing to variation.

Another potential factor contributing to intraspecific variation in aposematic signals is the interaction between selection by predation and sexual selection [13]. Honest sexual signals are likely to exhibit variation because they reflect underlying variation in mate quality [14], and sexual selection may act in opposition to predator selection for uniformity [15]. Identifying the proximate causes of color production can reveal potential costs and constraints, and suggest aspects of the information content of signals. For example, structural coloration is produced by submicron-level organization creating constructive interference with light [16]. Quality and regularity of the ultrastructure can affect its visual qualities [17]. Structural colors can be sensitive to perturbations during development, so poor-quality patches may indicate developmental stress [18], or poor genetic regulation [19]. Pigments are light-absorbing molecules that do not necessarily require fine-scale organization, but can also be sensitive to developmental stress [20], and may reveal different information to structural color [21]. Two well-studied examples are carotenoids, which produce red or yellow coloration and are involved in antioxidant defense [22], and melanin, which produces brown and black coloration and is an integral component of the insect encapsulation response to parasites [23]. Some pigments can only be sequestered from diet, while others may be costly to manufacture *de novo*, so different types of pigment can relate to different aspects of mate quality [24]. Variation in color patterns may therefore be informative to mate choice and may be under strong sexual selection and/or physiological constraints, counteracting predator selection for uniformity of aposematic signals.

In addition to selection by predators, sexual selection, and physiological trade-offs, elements of color patterns may be sensitive to environmental factors. Examples include temperature-induced melanization [25] [26], and effects of food limitation on carotenoid intake [22] or nitrogen intake necessary for pteridine synthesis [27] [28]. Such ‘direct’ costs may induce variation irrespective of the ‘indirect’ costs of sexual or predator selection. Therefore, we suggest that a ‘bottom-up’ approach of investigating color production mechanisms is a productive avenue of research for identifying the potential selective pressures, trade-offs, and constraints that may be shaping color patterns.

**Figure 1:**
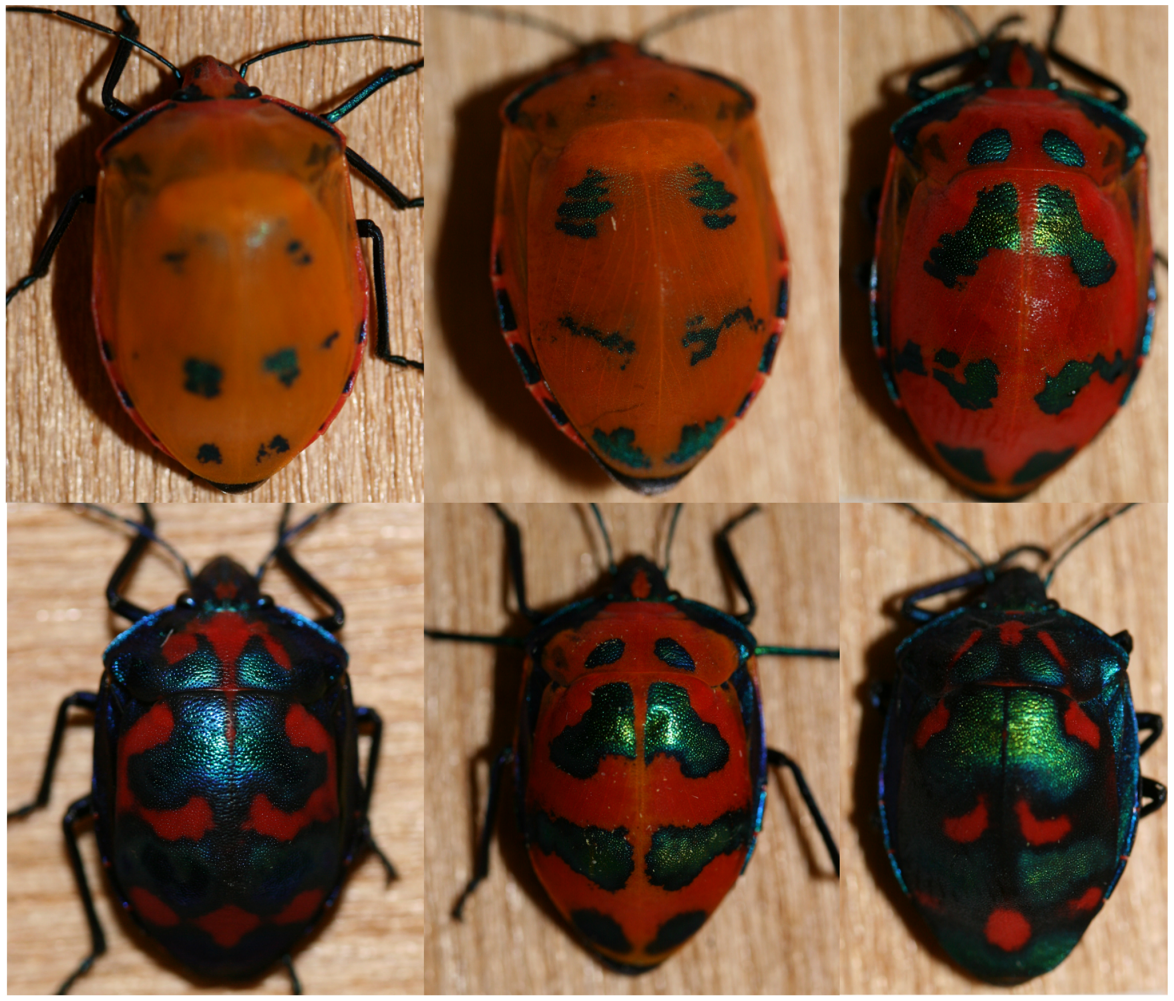
Examples of variation among individual of *T.* *diopthalmus*. Pictured are six individual *T. diopthalmus*; the top row is females, the bottom row is males, as examples of the variation between individuals in *T. diopthalmus*. This is not the extent of possible variation, as both males and females are capable of displaying patterns between complete absence and near total coverage of iridescence, and between rich red and virtually white pale orange.

In this study, we characterize and quantify the color production mechanisms of *Tectocoris diopthalmus* (Heteroptera: Scutelleridae), the Hibiscus Harlequin Bug. This large, charismatic stinkbug is widely distributed along the eastern and northern coasts of Australia and nearby Pacific islands [29]. Its defensive secretions have been identified [30], and these chemicals are known to be aversive to some predators, including birds [31] and praying mantids [32]. Rather than employing the more ‘typical’ aposematic color scheme of red or yellow with black markings, *T. diopthalmus* display a matte red-orange background with bright metallic blue-green iridescent patches. Because of their iridescence (the phenomenon of observed hue changing with viewing angle), these patches are likely to be produced by structural coloration. Both pattern elements are highly variable; the base color varies from a saturated red to very pale orange, while the iridescent patches range in hue from violet to green, and range in size from almost covering the dorsal surface to being entirely absent (figure 1). The species is sexually dichromatic, with males more likely to have large iridescent patches and deeper red coloration [33]. There are broad latitudinal [34] and seasonal [33] patterns in variation, as well as variation between individuals in one population at a given time. The use of iridescence in aposematic patterns is somewhat surprising, because the hue shifts with viewing angle introduce even further variability to the pattern (but see [35] and [36] for other potential cases of iridescent aposematic signals). The prominent use of both putatively structural and pigmentary color make *T. diopthalmus* an ideal candidate for detailed investigation. Furthermore, *T. diopthalmus* is sympatric over part of its range with *Cantao parentum*, a similar-sized scutellerid with similar life-history that displays a more ‘conventional’ red with black spotted pattern [29], which raises the question of why *T. diopthalmus* in particular features iridescent patches.

Our aim is to use histological and chemical methods to examine the color production mechanisms of *T. diopthalmus*, and thus the proximate causes of color variation in a putatively aposematic, sexually dimorphic bug. Characterizing the mechanisms will also facilitate identifying potential physiological costs and help elucidate information content of the various signal components. Furthermore, identification of color mechanisms will highlight specific avenues for future research into factors maintaining color variation, and is an important step for exploring the paradox of intraspecific variation in aposematism.

## Materials and Methods

Adult male and female Hibiscus Harlequin Bugs (*Tectocoris diopthalmus*) were collected off Norfolk Island Hibiscus (*Lagunaria patersonia*) trees planted on streets near the beaches of Narrabeen and Dee Why, New South Wales, Australia. No specific permissions were required to collect insects from local council-planted trees, and this species is not considered threatened or otherwise protected under law. Bugs were maintained in the lab on potted Beach Hibiscus (*Hibiscus tileaceus*) plants supplemented with seed pod cuttings from Norfolk Island Hibiscus and Native Rosella (*Hibiscus heterophyllus*) plants. Individuals were killed with ethyl acetate fumes, as freezing appeared to alter the appearance and likely the ultrastructure of the iridescent patches.

To quantify the color of the iridescent and orange patches, reflectance spectra of bug integument (12 males, 10 females) were obtained using an Ocean Optics USB 4000 Spectrophotometer with a fiber optic probe positioned at a 45° angle to the incident light source. The light source was an Ocean Optics PX-2 Pulsed Xenon lamp with an optic fiber positioned above the specimen. Insects were pinned to a three-axis freely rotating stage and rotated to positions of maximum brightness reflected. Samples were measured between 300 and 700 nm. Polytetraethylene (Teflon™) tape was used as the white standard, which provides over 97% reflectivity over the spectral range of interest [37]. To test for linearly polarized reflectance, specimens were viewed through a Hoya linear polarizer while rotating it; to test for circularly polarized light, specimens were viewed through a Hoya linear polarizer rotated behind a quarter-wave plate [38].

Electron microscopy was used to image the cuticular ultrastructure responsible for creating the iridescent patches. One representative male and one female were imaged by Scanning Electron Microscopy (SEM). Pieces of the scutellum were fixed in a 3% glutaraldehye solution in phosphate buffer pH 7.2 overnight, dehydrated in a graded ethanol series (50%, 70%, 90%, 100%, 100%; 15 minutes each step), and dried with an Emitech K850 critical point dryer. The pieces were then mounted on aluminium stubs and sputter coated with gold (approximately 20 nm thick). Specimens were viewed with a Jeol JSM 6480 scanning electron microscope.

Two males, one female, and one juvenile bug were imaged for Transmission Electron Microscopy (TEM). Pieces of scutellum were fixed in a 3% glutaraldehyde solution in phosphate buffer pH 7.2 overnight, and post-fixed in a 1% osmium tetroxide solution for 2 hours, followed by a 2% uranyl acetate solution for 30 minutes. The samples were dehydrated using a graded ethanol series (50%, 70%, 80%, 90%, 95%, 100%, 100%; 30 minutes each step), and infiltrated in LR White resin using a resin-ethanol series (1∶3, 1∶2, 1∶1, 2∶1, 3∶1, 100% ×2, 1.5 hours each). The final immersion was held under vacuum for 5 hours, before being loaded into molds and polymerized at 70°C. Semi-thin (0.7 µm) and ultra-thin sections (∼70 nm) were cut perpendicular to the dorsal surface using a Reicherts Ultracut S microtome with a diamond knife. Semi-thin sections were viewed with an Olympus BH-2 microscope with Scion CFW-1310C color digital camera. Ultra-thin sections were mounted on copper TEM grids coated with 0.3% pioloform, and stained with 7.7% uranyl acetate for 30 minutes, followed by Reynolds lead citrate [39] for 5 minutes. Specimens had a sectioned transversely across the color interface. Sections were imaged with a Philips CM10 transmission electron microscope with Olympus SIS Megaview G2 digital camera. Measurements of layer width were made at six random points along the transverse section using Olympus iTEM 5.1 software.

For preliminary pigment analysis, carotenoid presence was tested using the acidified pyridine method [40]. To distinguish between other candidate pigment classes (melanins, pterins, flavonoids, and ommochromes), a discriminatory extraction test, modified from Lindstedt et al. [41], was used. Pterins and flavonoids are soluble in strong acids and bases, but flavonoids are also soluble in neutral organic solvents such as methanol, while ommochromes are soluble in acidified alcohols [28] [42]. Both intact and crushed-whole bugs were placed in vials of 0.1M sodium hydroxide, 90% methanol, or 1∶10 hydrochloric acid/methanol solution, and incubated at room temperature for 24 hours. Insect parts were removed and the extracts were centrifuged at 14,000 RPM for 5 minutes. The supernatants were then measured using a Shimadzu UVmini 1240 UV-Vis Spectrophotometer using quartz cuvettes across a wavelength range of 200 to 800 nm. Absorbance spectra were compared against published spectra [43]. Both intact bugs and extracted supernatants were observed under short-wave (254 nm) and long-wave (366 nm) ultraviolet light to visually inspect for the presence of fluorescence, as pterins and flavonoids fluoresce under UV light while carotenoids, ommochromes, and melanins do not [28] [42].

Based on the preliminary results of pigment analysis, we focused our identification efforts on pterin pigments. For the separation and identification of pterins potentially present in the integuments of *T. diopthalmus*, a capillary electrophoretic method (CE) was developed. The separation system was modified from Han et al. [44]. The CE measurements with UV detection were carried out on an Agilent Technologies HP^3D^CE system with built-in diode array detector operated at 250 nm. CE analyses were conducted in an uncoated fused-silica capillary (CACO) total length 70 cm, 55 cm to the detector, inner diameter 50 µm, thermostated at 30°C. Background electrolyte (BGE) contained a mixture of 100 mmol/L boric acid, 100 mmol/L tris(hydroxymethyl)aminomethane (TRIS), pH 9,0, and 2 mmol/L ethylenediaminetetraacetic acid disodium salt dihydrate (Na_2_EDTA). Samples were injected electrokinetically at 20 kV for 10 seconds, and the applied separation voltage was 20 kV. Pterin standards included biopterin, isoxanthopterin, leucopterin, neopterin, xanthopterin, and erythopterin (all from Sigma Aldrich except erythropterin provided by R. Rutowski). Stock solutions of the individual standards were prepared by dissolving the compounds in dimethyl sulfoxide at a concentration of 0.1 mg/mL and were kept in dark at 4°C. Working standard solutions were prepared by diluting the stock solutions with BGE to a concentration of 0.025 mg/ml. Identification was carried out by spiking samples with pure standard solutions.

Dried integuments were used in the CE analysis. Three bugs of each color morph (‘red’ vs ‘orange’, as judged visually by experimenter) were used in each extraction, to minimize the effects of individual variation. Integuments were weighed (∼6 mg per red extract and ∼8 mg per orange extract) and put in a vial with 0.5 mL dimethyl sulfoxide to be incubated in the dark at room temperature for 98 hours. The extract was centrifuged at 13,000 RPM for 10 minutes. The supernatant was then diluted 4× with BGE before being used for CE analysis. Three extracts of red form and orange form were prepared, for a total of nine individuals sampled per morph.

To test whether the otherwise-insoluble iridescence is dependent on melanin for its optical properties, photographs and spectra of 5 sample bugs were taken before being immersed in 20% hydrogen peroxide for 24 hours, after which the bugs were washed with water, photographed and measured spectrally (see above) again. Hydrogen peroxide breaks down melanin [45], and degrade melanin-containing layers in the ultrastructure in situ, decreasing both peak reflectance wavelength and brightness [46].

## Results

**Figure 2:**
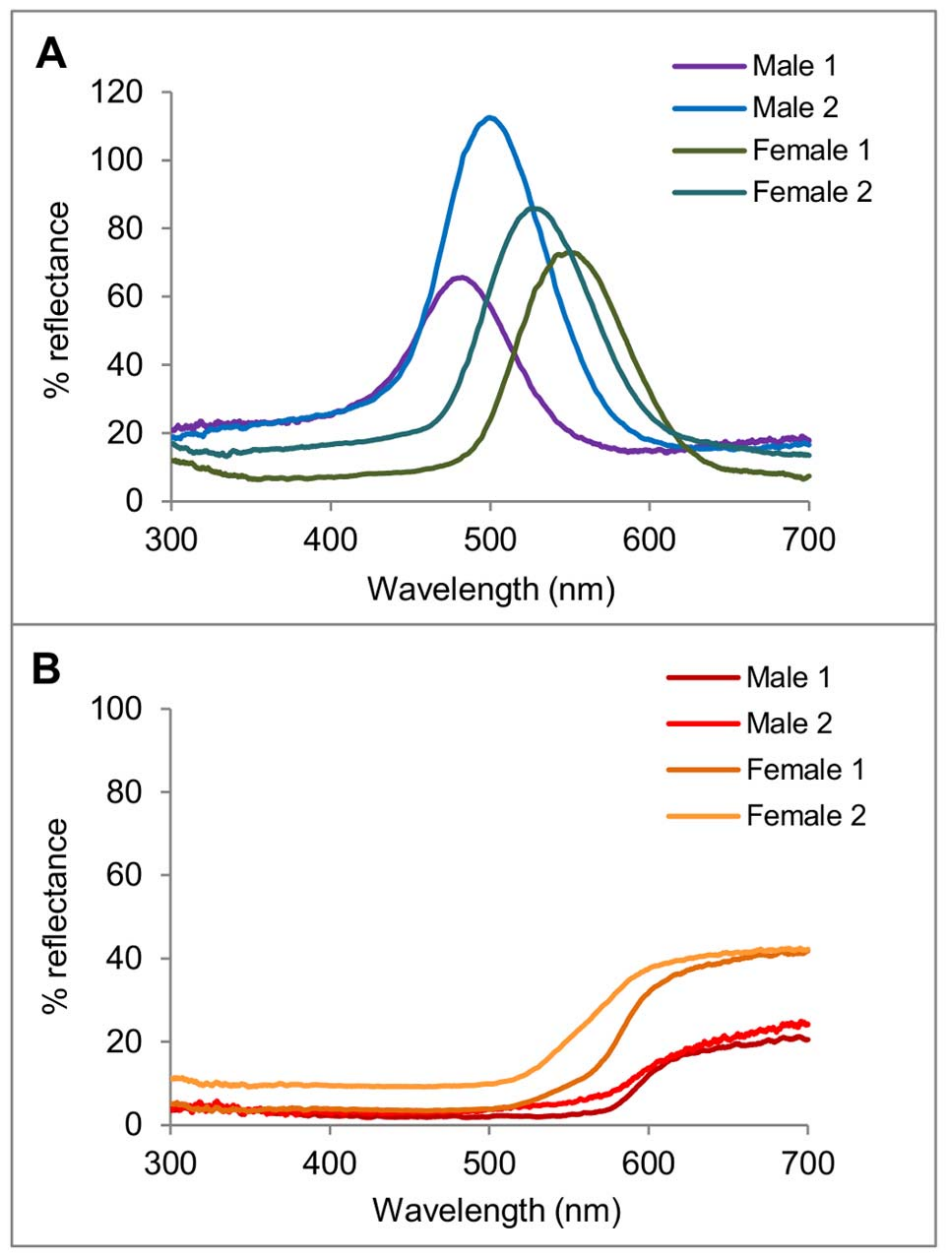
Example reflectance spectra of *T.* *diopthalmus* color patches. The spectral reflectance curves of the color patches of four individuals (two males, two females) are shown here. Curves from iridescent dorsal patches are shown in A, while the curves from the dorsal orange patches of the same individual are shown in B. Precent reflectance is against a Teflon white standard.

Selected example spectra for the iridescent and orange patches can be seen in Figure 2. The iridescent patches show a sharp peak, which, in the individuals sampled, can range between 480 and 570 nanometers, while the orange base shows a monotonic increase beginning at between 512 and 590 nanometers, leveling off by 700 nanometers. In this study male scutellum iridescent patches are on average more blue-shifted (average peak of 520±24 nm versus 542±17 nm for females), while the male's orange patches are more red-shifted, reflecting on average light of 580±14 nm or greater (versus 538±24 nm or greater for females), creating greater chromatic contrast. No polarized reflectance, either linear or circular, was detected at normal light incidence and viewing angle in either sex.

**Figure 3:**
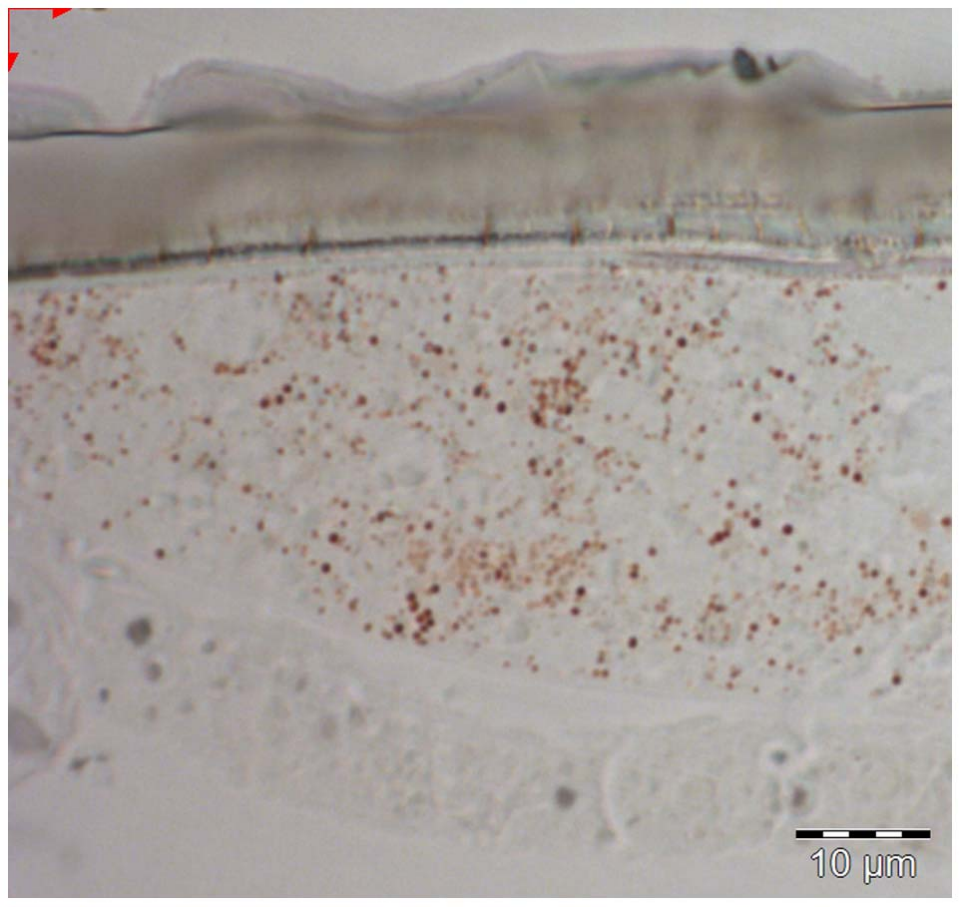
Light microscopy image of epidermal cell in *T.* *diopthalmus*. Unstained thin section of an epidermal cell underneath cuticle in the ventral surface of *T. diopthalmus*. The small red granules near the distal end are believed to be pigment granules containing pterins.

In the differential solubility test, an orange-colored pigment was soluble in 0.1M NaOH and acidified methanol, but not neutral methanol (Table 1). The absorbance peak in acidified methanol was at 430 nm, with a trough at 380 nm, closely approximating published spectra for erythropterin [43]. Extracts could not be obtained from intact bugs, but only from ground specimens. Unstained semi-thin sections of cuticle reveal red granules inside epidermal cells underneath unpigmented cuticle (Figure 3). Both pigment extracts and the venters of intact bugs fluoresce under ultraviolet light (Table 1).

**Table 1:** Discriminatory identification of pigment class.

	Dissolves in solvent				Fluoresces in UV
Pigment Class	Acidified Pyridine	0.1M NaOH	90% Methanol	10:1 Methanol/HCl	
Carotenoids	X				
Pterins		X		X	X
Flavinoids		X	X	X	X
Ommochromes				X	
*T. diopthalmus* extract		X		X	X

X indicates that the pigment named in the row is readily soluble in the solvent named in the column, or fluoresces under ultraviolet light. Lack of X indicates no visible extraction or fluorescence.

**Figure 4:**
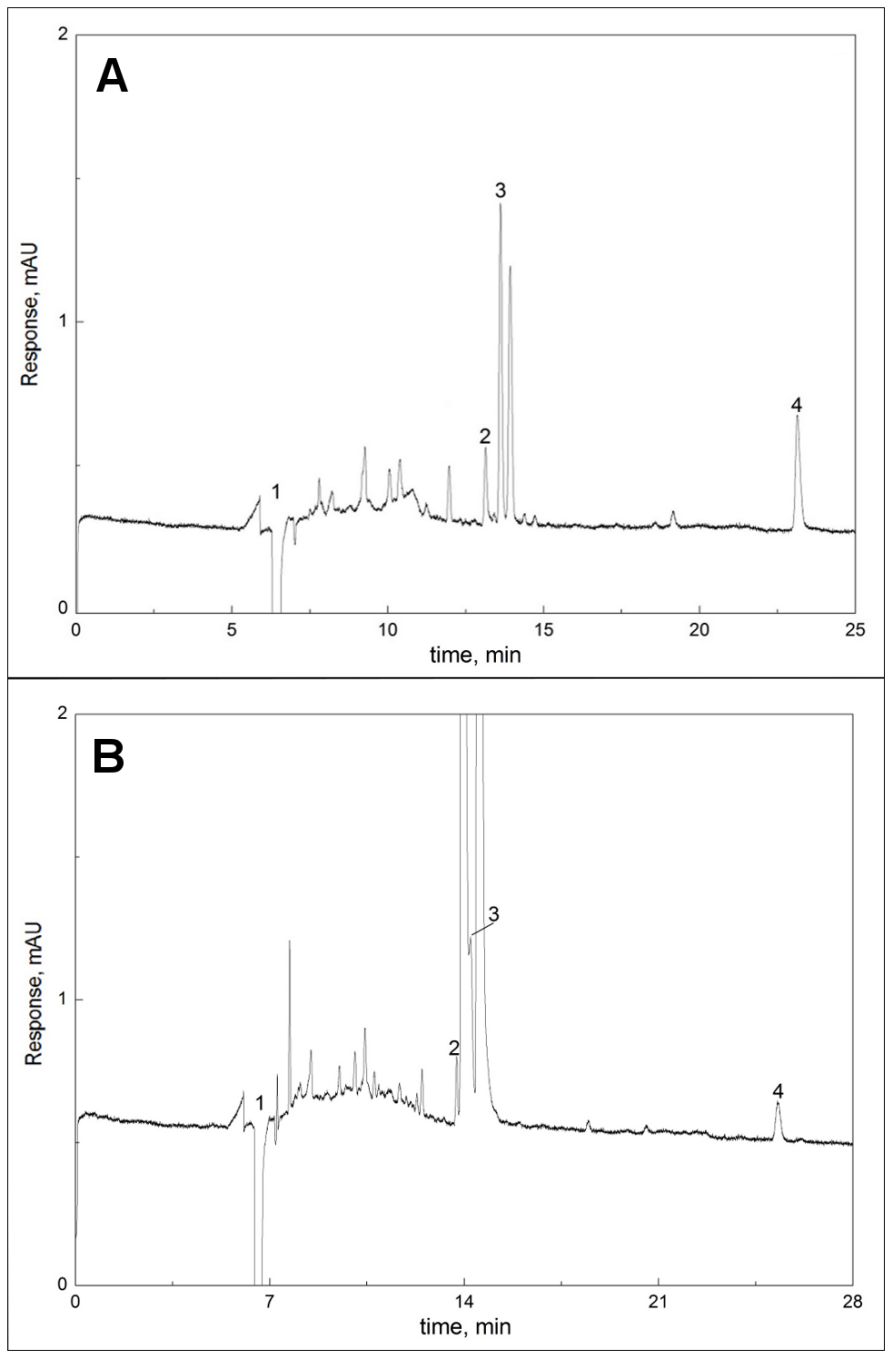
Analysis of pterin-based pigments of *T.* *diopthalmus* by Capillary Electrophoresis (CE). Capillary Electrophoresis (CE) analysis of red (A) and orange (B) forms of integuments of *Tectocoris diopthalmus* at 250 nm. Peaks: 1- dimethyl sulfoxide,2- isoxanthopterin, 3-leucopterin, 4-erythropterin. Note the larger erythropterin peak in the red form.

For confirmation and specification of pterin derivative(s) responsible for the coloration, organic extracts of integuments from orange and red morphs of *T. diopthalmus* were analyzed by capillary electrophoresis (CE). The results obtained from one extraction of ‘red’ and ‘orange’ forms are shown in Figure 4. Four peaks were identified in both morphs. Peak 1, the negative peak, is due to dimethyl sulfoxide in the injected sample. Peaks 2 and 3 correspond to isoxanthopterin and leucopterin respectively, which are colorless in visible wavelengths but absorb ultraviolet light. Peak 4 is erythropterin, a red-reflecting pigment which absorbs shorter wavelengths. Both morphs also contain small amounts of colorless biopterin [unlabeled]. Qualitatively, electrophoreograms obtained for a given color morph were nearly identical. The key color difference identified between red and orange morphs is the greater amount of erythropterin present in the red morph (mean peak area 4.23±0.59 milliabsorption units*seconds, from 6 mg of integument) versus the orange morph (mean peak area 2.87±0.15 milliabsorption units*seconds, from 8 mg of integument). Xanthopterin, a common reddish-orange pigment, was not found in any tested samples.

**Figure 5:**
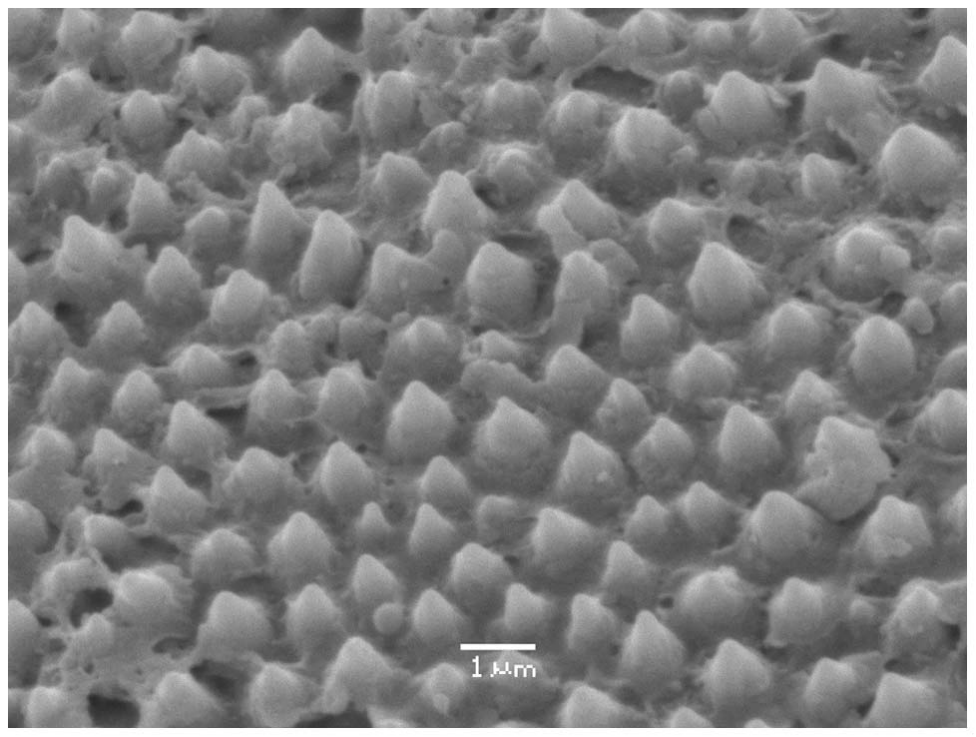
Scanning Electron Microscopy (SEM) image of scutellum surface. SEM imaging shows conical tubercles of varying dimensions covering the surface of the scutellum. These protrusions can be found on both males and females and overlying both iridescent and orange patches.

Scanning electron microscopy revealed that the dorsal surface is covered by microtubules of uneven size but roughly even spacing. These microtubules can be found uniformly over the scutellum of both males and females regardless of underlying color (Figure 5). Transmission electron microscopy revealed no microstructure in the cuticle of the orange patches.

**Figure 6:**
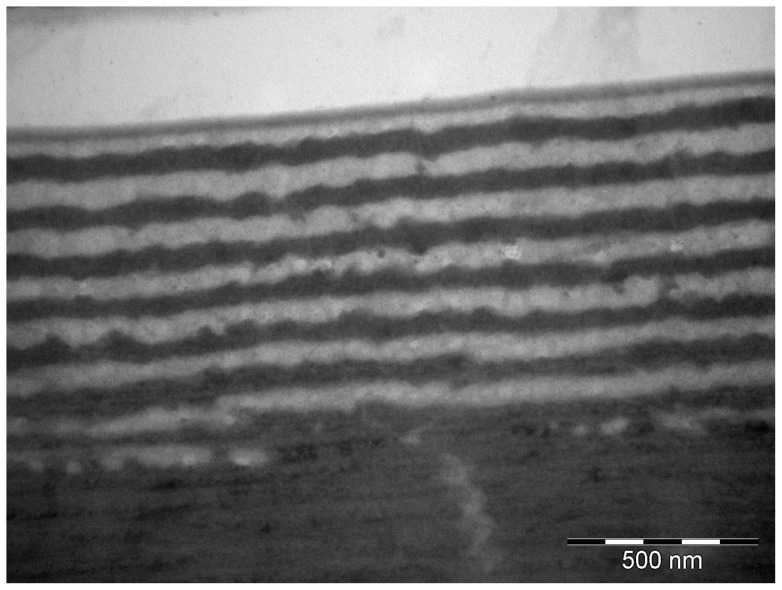
Transmission Electron Micrograph (TEM) of the multilayer reflector. TEM image of the multilayer reflector in *T. diopthalmus* (from sample 2 in table 2). Note the bottommost thin ‘dark’ layer appears contiguous with the underlying thick dark layer where the separating ‘light’ layer is disrupted.

**Figure 7:**
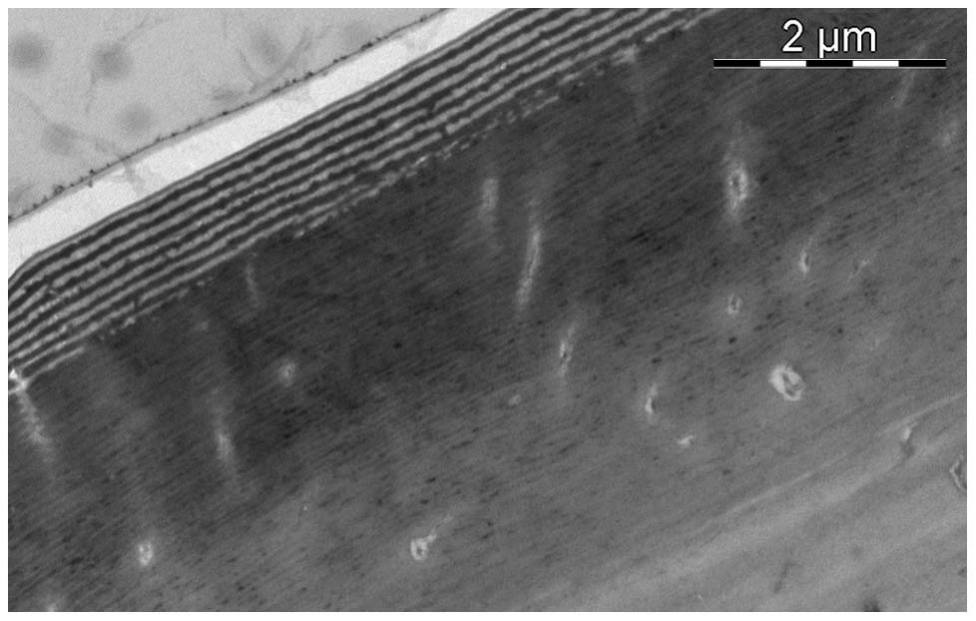
Transmission Electron Micrograph (TEM) of Epicuticle and Exocuticle. Zoomed out version of Figure 6. Three regions of cuticle can be seen. The thin banding in the top-left region is the epicuticle, the outermost layer of cuticle, containing the multilayer reflector. Below that is the exocuticle, with fine helicoidal layering. The upper region of the exocuticle contains an electron-dense pigment which appears brown in unstained thin sections for light microscopy (not pictured). The smoother region in the bottom-right corner is the upper edge of the endocuticle. The apparent thick white layer in the upper-right corner of the micrograph is an artefact due to resin separation from the sample.

TEM imaging did however reveal ultrastructure to the iridescent patches compromising a multilayer system in the epicuticle (Figure 6). The structure consists of alternating layers of ‘dark’ (electron-dense) and ‘light’ (electron-lucent) material, bordered on the outside by a thinner semi-lucent layer, and underlaid by a thick layer of pigmented exocuticle (Figure 7). There are between 5 and 9 layers, on average 7, of each layer type. The average width of the layers is between 60 and 90 nanometers, depending on the individual measured (Table 2). At the base of the multilayer system, and at the interface between the iridescence patches and the black border regions (not shown), the ‘light’ layers degenerate, and the ‘dark’ layers seamlessly merge with the underlying pigmented layer. The underlying layer appears as a thick brown line under light microscopy. For a comparison of measured and predicted peak wavelengths of the iridescent patches in the individuals examined, see Table 2.

**Table 2:** Multilayer reflector layer measurements and predicted peak reflectance.

Sample	Age/Sex	Measured peak reflectance (nm)	Number of ‘dark’ layers	Width of ‘dark’ layers (nm)	Number of ‘light’ layers	Width of ‘light’ layers (nm)	Predicted peak reflectance (nm)
1	Male	462	7.2±1.2	59.2±5.9	7.3±0.8	70.3±4.5	456±21
2	Male	532	6.8±1.5	73.1±5.9	6.5±1.3	79.4±6.3	540±21
3	Female	538	6.2±1.3	68.6±9.8	5.8±0.4	90.6±5.9	557±34
4	Juvenile	463	4.8±0.4	60.9±4.6	5.2±0.4	64.3±5.7	444±19

Layer widths (and resulting predicted peak reflectance) given are the mean and standard deviation of the average layer width at six points along the section; therefore, standard deviations reflect horizontal regularity rather than vertical. For examples of ‘dark’ and ‘light’ layers, see Figure 6.

**Figure 8:**
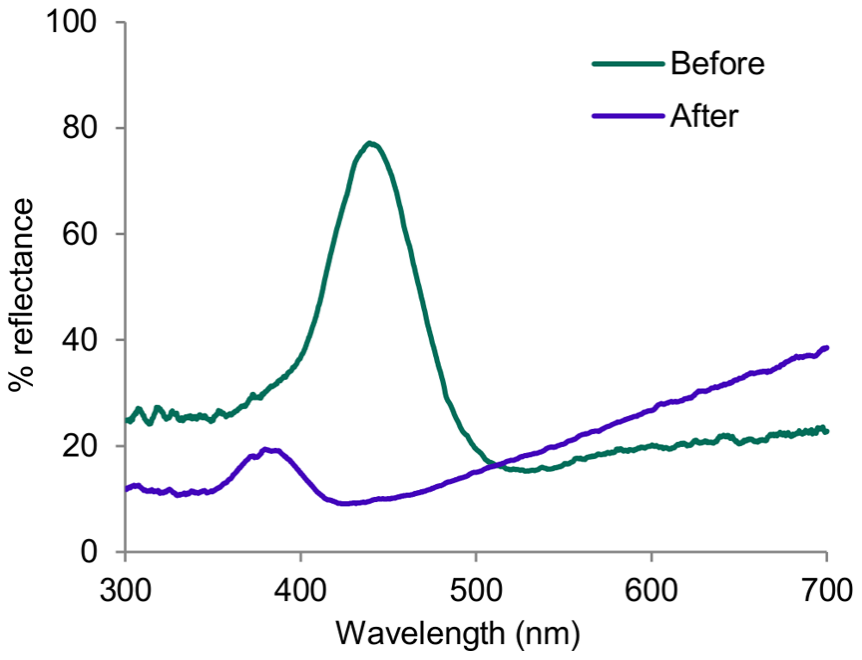
‘Before’ and ‘After’ reflectance spectra after soaking bug in 30% hydrogen peroxide. ‘Before’ is the reflectance spectrum of the bug before soaking 24 h in 30% H_2_O_2_, ‘After’ is the spectra taken after drying. Percent reflectance is against a Teflon white standard. Note the peak shifting to shorter wavelengths, reflective of the thinning of the layers in the multilayer system, and the monotonic increase at longer wavelengths.

For the bugs soaked in hydrogen peroxide, the peak reflectance of the iridescent spots was shifted to a shorter wavelength, the peak brightness was dramatically reduced, and the broadband reflectance was increased, with a monotonic rise at longer wavelengths (Figure 8). This is indicative of both a marked reduction in the optical thickness of the layers and leeching of the underlying pigment.

## Discussion

### Identification of color production mechanisms

We identified two major color production mechanisms in *Tectocoris diopthalmus*, a red-orange pigment and a multilayer reflector structure. The structure exists in the outermost exoskeletal layer, while the pigment is contained in intracellular granules in the epidermis. We identified the pigment as a pterin-based compound due to its fluorescence characteristics and solubility in acidified or basic aqueous or organic solvent (Table 1). The color causing pigment has been specifically identified as erythropterin by capillary electrophoresis, and differences in the hue of the red-orange coloration can be largely explained by differences in the amount of erythropterin pigment. This result agrees with previous studies that have identified erythopterin as a major red or orange pigment in heteropterans [47] [48] [49] [50].

The iridescent blue-green coloration is produced by a multilayer reflector in the epicuticle, identifiable in transmission electron micrographs (TEM). Both the structure and results of hydrogen peroxide bleaching closely match the results of Schultz and Rankin [46], thus the Harlequin Bug and *Cicindela* tiger beetles have likely evolved a convergent system. We therefore posit that the multilayer structure in *T. diopthalmus* is also is composed of alternating layers of melanin-laced and melanin-free protein matrix, overlaying a melanized exocuticle base [46]. These iridescent patches could be an elaboration evolved directly from melanic patches in a more ‘typically aposematic’ red and black ancestor, perhaps in shared ancestry with *Cantao*, but this hypothesis requires phylogenetic analysis for further investigation.

The ultrastructure of the epicuticular reflector appears similar to those found in other non-aposematically colored scutellerids [51] [52], as well as cicindelid tiger beetles [53], buprestid jewel beetles [54], and calopterygid damselflies [55]. This is structurally distinct from the rotated helicoidal rotated exocuticle found in scarab reflectors [16], as well as the epicuticular reflector in wasps [56]. The function of iridescence in these animals is often unknown, though it is believed to aid crypsis in *Cincindela oregona* tiger beetles [57] while functioning as a sexual trait, as an indicator of male condition, in male *Calopteryx maculata* damselflies [55].

The predicted peak reflectance of the multilayer system can be calculated using Snell's Law, 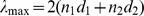 wherein the peak reflectance (λ_max_) is dependent on the refractive index (n) and thickness (d) of each layer type. Based on measurements of peak reflectance and layer widths, the average refractive index is estimated to be 1.78. Durrer and Villiger [58] estimated the average refractive index of a melanin-based epicuticular reflector to be 1.75, with the melanic layers having a refractive index of 2.0 and non-melanic layers having an index of 1.5. This estimate has been used successfully by Schultz and Rankin [46] and Fitzstephens and Getty [55] to predict the peak reflectance of multilayer systems. More recent studies by Noyes et al. [59] and Stavenga et al. [54] place the estimate for melanic layers lower, at between 1.6 and 1.7. Using these lower estimates results in a predicted peak wavelength far lower than the value we measured. We therefore utilize the higher value of 2.0 as the refractive index of our electron-dense layer, and consequently 1.56 for our electron-lucent layer. However, we acknowledge the limitations of our analysis and do not intend to make broad claims as to the optical nature of ultrastructural components. Furthermore, diverse claims of the refractive index of ultrastructure components have been made in other scutellerids, including chitin and water (1.58 and 1.33) in *Poecilocoris lewisi* [51], and chitin and chitin-air grating (1.56 and 1.4) in *Calidea panaethiopica* [52]. Therefore there may be great evolutionary lability and diversity in ultrastructural components.

The outer surface of the scutellum in both sexes is uniformly covered by small bumps visible in scanning electron micrographs. These microtubercles appear superficially similar to structures found in another scutellerid, *Poecilocoris lewisi*, which produce a diffuse blue reflectance via Mie scattering [51]. Curiously, we see no evidence for a similar effect in reflectance spectra of *T. diopthalmus* (Figure 2), and do not believe the microtubercles function in color production. It may be that these protrusions act as an anti-reflector structure to reduce specular reflectance, but their dimensions are too large to function effectively in this capacity [60]. Alternatively, they may not have any visual function, and act as an anti-wetting structure [60]; further studies are required to elucidate their biological function.

### Exploring the implications of identified mechanisms

The red-orange color is created primarily by erythropterin pigment. Pterins are endogenously produced, though they are some of the most nitrogen-heavy pigments [28] [61]. It has been proposed that pterins are used as a nitrogenous waste product in insects, suggesting the pigment is ‘cheap’ [62]. However, plant herbivory often results in nitrogen limitation as an important factor in life history trade-offs [27]. Variation in pterin pigmentation between the sexes and between individuals could reflect differences in the ability to acquire dietary nitrogen, but variation in color can be found between individuals reared on the same host plant (personal observation). While the role of pterins as antioxidants in insects has not to our knowledge been studied, certain pterins are known to have antioxidant and immune modulating functions in vertebrates [63]. Given the prevalence of pterins as heteropteran pigments [47], we suggest that the use of erythropterin is as a phylogenetically conserved ‘cheap’ pigment for aposematic function, but whether it has protective function in Heteroptera requires further study. Furthermore, the average shift in reflectance between males and females suggests that the red-orange pigment may also be playing a role in the creation of a sexual signal in addition to any aposematic function. Selective forces such as mate choice and predators may be selecting on the color pattern as a whole, so it is unsurprising that an element of the color pattern is likely contributing to multiple functions.

As a potentially ‘derived’ element of the color pattern, we are particularly interested in how the multilayer reflector system in the iridescent patches may act as an informative, honest sexual trait. It is more structurally complex than the pigmentary component of the signal. In addition to patch size and symmetry, signal quality depends on the regularity of both the dark and light layers, in both the vertical and horizontal dimensions. The precision required for this structure may be disturbed by developmental instability, making it a sensitive trait to display genetic quality and resistance to perturbation [19]. Pigmentary colors can also be sensitive to rearing conditions, and the reliance on melanin pigment as a major component may make the patches even more sensitive to environmental disturbance. For example, cuticular melanization can be upset by high temperatures in many taxa including Lepidoptera [64], *Drosophila* flies [26], and other Heteroptera [25]. This can help explain population differences, with high ambient temperatures suppressing the patches in tropical populations [34], and depending on the window of susceptibility, can also be contributing to intrapopulation variation, via seasonal or daily fluctuations in temperature [33].

Melanin-based signals may also be informative through trade-offs with physiological uses for melanin and its precursors, in ways unique to invertebrates [65]. Tyrosine limitation may result in trade-offs with production of neurotransmitter dopamine or cuticle hardening agent sclerotin [23] [66]. Moreover, melanin and its precursors are utilized in the encapsulation response to endoparasites, a vital component of insect innate immunity, and cuticular coloration could trade off with immunocompetence [67] [68]. Conversely, enhanced melanic ornamentation could also be a result of greater systemic levels of melanin precursors or enzymes. In this way, the iridescent patches could act as an honest indicator of condition or immunocompetence [69]. Juvenile immune challenge can also influence adult expression of melanin coloration, rendering it an informative artefact of juvenile infection history [70].

### Variation and its effects on aposematism

Despite the inherent variability in the iridescent patches, there may be aposematic benefit in iridescence. The ‘complimentary’ nature of green/blue and red/orange (e.g. little overlap in reflected wavelengths) enhances conspicuousness compared to a red/black pattern of comparable brightness [71]. The patches are also highly reflective and saturated, and the juxtaposition of bright and chromatic patches with a low brightness and chroma background (e.g. the red-orange base color) also enhances signal conspicuousness [71]. This brightness contrast may be especially important in aposematism against invertebrate predators such as praying mantids, whose hunting tactics may be more reliant on luminance contrast [72]. The downside of having iridescence is its variability, as there is increased predation risk for individuals that do not match the common morph [4] [73]. However, Ihalainen et al. [74] found evidence that being variable may in fact be beneficial to avoidance learning of moderately defended prey. *Tectocoris diopthalmus*' defensive capacity is moderate compared to other heteropterans [30] [75], and thus may benefit from this variation.

Conversely, variability in hue of orange pigmentation may have little influence on aposematic defense. Exnerová et al. [76] demonstrated that four bird species showed no difference in avoidance learning between red and orange morphs of the firebug *Pyrrhocori apterus*. Great Tits (*Parus major*) trained to avoid red artificial prey will generalize to avoid orange prey as well [77]. Lindstedt et al. [78] showed that while birds in the lab can discriminate between red and orange morphs and will preferentially attack orange, ‘survival’ of models in the field was equal between morphs. With bugs of varying red and orange hues coexisting, birds may quickly learn to generalize. Variation in the size of iridescent patches has the potential for consequences on aposematism, as larger pattern elements increase avoidance in naïve chicks [79].

In conclusion, investigation into the mechanisms of color production has opened up a wealth of possible physiological and environmental interactions, and generated specific hypotheses to fuel future research directions. We have only used one species as a case study, but the commonplace chemical and microscopic techniques used here are easily applicable to many species. Variation in aposematism is a longstanding question in behavioral ecology, and a bottom-up approach of studying prey physiology is one way to help answer it.
