# Correction: Mechanisms of Color Production in a Highly Variable Shield-Back Stinkbug, *Tectocoris diopthalmus* (Heteroptera: Scutelleridae), and Why It Matters

**DOI:** 10.1371/annotation/416a5460-d2e9-4d5b-87fa-c6c8659f002d

**Published:** 2013-10-24

**Authors:** Scott A. Fabricant, Darrell J. Kemp, Jan Krajíček, Zuzana Bosáková, Marie E. Herberstein

The term "*diophthalmus*" was spelled incorrectly in the title at multiple points throughout the article.

In addition, in the last sentence of the legend for Figure 2, the word "percent" was spelled incorrectly.

